# Evaluation of an Artificial Intelligence and Online Psychotherapy Initiative to Improve Access and Efficiency in an Ambulatory Psychiatric Setting: Évaluation d’une initiative de psychothérapie en ligne basée sur l’intelligence artificielle visant à améliorer l’accès et l’efficacité en milieu psychiatrique ambulatoire

**DOI:** 10.1177/07067437251355641

**Published:** 2025-08-01

**Authors:** Callum Stephenson, Jazmin Eadie, Christina Holmes, Kimia Asadpour, Gilmar Gutierrez, Anchan Kumar, Jasleen Jagayat, Charmy Patel, Saad Sajid, Oleksandr Knyahnytskyi, Megan Yang, Taras Reshetukha, Christina Moi, Tricia Barrett, Amirhossein Shirazi, Vedat Verter, Claudio N Soares, Mohsen Omrani, Nazanin Alavi

**Affiliations:** 1 Department of Psychiatry, 4257 Queen's University, Kingston, ON, Canada; 2 OPTT Inc., Toronto, ON, Canada; 3 Centre for Neuroscience Studies, 4257 Queen's University, Kingston, ON, Canada; 4 71459 Kingston Health Sciences Centre, Kingston, ON, Canada; 5 Smith School of Business, 4257 Queen's University, Kingston, ON, Canada; 6 4256 Providence Care Hospital, Kingston, ON, Canada

**Keywords:** digital health interventions, mental health, e-mental health, mental health services, psychotherapy, artificial intelligence, natural language processing, triage, psychiatry, cognitive behavioural therapy, quality improvement

## Abstract

**Objectives:**

This study aimed to implement an artificial intelligence-assisted psychiatric triage program, assessing its impact on efficiency and resource optimization.

**Methods:**

This project recruited patients on the waitlist for psychiatric evaluation at an outpatient hospital. Participants (*n* = 101) completed a digital triage module that used natural language processing and machine learning to recommend a care intensity level and a disorder-specific digital psychotherapy program. A psychiatrist also assessed the same information, and the decisions for care intensity and psychotherapy programs were compared with the artificial intelligence recommendations.

**Results:**

The overall wait time to receive care decreased by 71.43% due to this initiative. Additionally, participants received psychological care within three weeks after completing the triage module. In 71.29% of the cases, the artificial intelligence-assisted triage program and the psychiatrist suggested the same treatment intensity and psychotherapy program. Additionally, 63.29% of participants allocated to lower-intensity treatment plans by the AI-assisted triage program did not require psychiatric consultation later.

**Conclusions:**

Using artificial intelligence to expedite psychiatric triaging is a promising solution to address long wait times for mental health care. With future accuracy refinements, this could be a valuable tool to implement in hospital settings to assist care teams and improve mental health care. This could result in increased care capacity and improved workflow and decision-making.

## Plain Language Summary Title

Evaluation of AI and Online Psychotherapy Initiative to Improve Psychiatric Care Access and Efficiency

Plain Language Summary

The mental healthcare system is overwhelmed by increasing patient demand, leading to long wait times for care. The needs of each patient vary based on their specific condition and the resources available. Digital health tools and artificial intelligence could address these challenges by streamlining care and matching patients to appropriate resources. This study explored using an artificial intelligence-assisted triage tool to improve the process of assigning care levels and treatment recommendations for patients waiting for psychiatric care. Researchers compared this artificial intelligence system to the decision-making of a psychiatrist. The study involved 101 patients from an outpatient psychiatry waitlist. Each participant completed a digital triage module using natural language processing and machine learning to recommend a care intensity level and suggest a digital psychotherapy program tailored to their needs. The psychiatrist also reviewed the same participants’ responses and compared the results. The findings were promising since they showed that the AI-assisted triage program reduced wait times by 71%, allowing participants to access care within three weeks of completing the module. Additionally, in over 70% of cases, the AI-assisted triage program recommendations for treatment intensity and psychotherapy matched those of the psychiatrist. Furthermore, 63% of patients who were allocated to lower-intensity care plans by artificial intelligence did not need further psychiatric consultation. This suggests that the tool effectively identified appropriate care pathways for many patients. These results highlight the potential of artificial intelligence in alleviating the burden on mental healthcare systems. By accelerating triaging and improving decision-making, these digital tools could increase care capacity and streamline workflows. With further refinement, this technology could be a game-changer for hospitals and mental health services.

## Résumé

**Objectifs:**

Cette étude visait à mettre en œuvre un programme de triage psychiatrique assisté par l’intelligence artificielle, afin d’évaluer son incidence sur l’efficience et l’optimisation des ressources.

**Méthodes:**

Ce projet a permis de recruter des patients inscrits sur la liste d’attente pour une évaluation psychiatrique en consultation externe. Les participants (*n* = 101) ont rempli un module de triage numérique qui utilisait le traitement automatique du langage naturel et l’apprentissage automatique pour recommander un niveau d’intensité des soins et un programme de psychothérapie numérique adapté au trouble. Un psychiatre a également évalué la même information, et les décisions relatives à l’intensité des soins et aux programmes de psychothérapie ont été comparées aux recommandations de l’intelligence artificielle.

**Résultats:**

Le temps d’attente global pour recevoir des soins a diminué de 71,43 % grâce à cette initiative. De plus, les participants ont reçu des soins psychologiques dans les trois semaines après avoir terminé le module de triage. Dans 71,29 % des cas, le programme de triage assisté par l’intelligence artificielle et le psychiatre ont suggéré la même intensité de traitement et le même programme de psychothérapie. De plus, 63,29 % des participants affectés à des plans de traitement de moindre intensité par le programme de triage assisté par l’IA n’ont pas eu besoin de consultation psychiatrique par la suite.

**Conclusions:**

L’utilisation de l’intelligence artificielle pour accélérer le triage psychiatrique est une solution prometteuse pour réduire les longs délais d’attente dans les soins de santé mentale. Avec des améliorations futures en matière de précision, cet outil pourrait s’avérer précieux dans les milieux hospitaliers pour aider les équipes soignantes et améliorer les soins de santé mentale. Cela pourrait se traduire par une augmentation de la capacité de soins, et une amélioration du flux de travail et de la prise de décisions.

## Introduction

Approximately 6.7 million Canadians experience mental health challenges annually, with half reporting unmet or partially unmet needs.^1–6^ This contributes to >50B/year^4,5^ of financial burden. The COVID-19 pandemic exacerbated the strain on mental healthcare systems, lengthening wait times, increasing emergency department (ED) visits, and raising costs.^1^ On average, Canadian patients wait seven months to begin psychiatric treatment.^7^ In the absence of timely outpatient care, many turn to EDs, resulting in a 50% rise in mental health-related ED visits in Ontario from 2011 to 2021.^8,9^ Nearly half of these visits are first contact, highlighting barriers to outpatient services.^10–12^ ED care is also costly, at $500–600/visit compared to $80–150/visit in outpatient settings.^8,13^ Furthermore, untreated mental health issues increase hospital stays, readmissions, and chronic disease management costs by 1.5–3 times.^14–20^

The Kingston-Frontenac-Lennox and Addington regions face similar challenges. Kingston Health Sciences Center (KHSC), which serves 650,000 people annually, had 1,200 individuals on its outpatient psychiatry waitlist and psychiatric wait times of 12–16 months in October 2023. In 2022, 9.02% of KHSC ED visits (4,760) were mental health-related, with 73.53% being redirected to outpatient care. Improving outpatient service access could have prevented a majority of these visits, saving $1.4 M/year and reducing ED demand by 6.63%.

To optimize resource allocation, Ontario employs stratified and stepped-care models, prioritizing psychiatrist appointments for severe cases.^21–24^ Current triage processes, such as those at the Mental Health and Addiction Clinic at Hotel Dieu Hospital (HDH), require a 45-min interview conducted by a registered nurse before they deliberate with the healthcare team and assign care. Although effective, this approach is resource-intensive and prolongs wait times.^21–24^

To improve care efficiency, the team developed an AI-assisted triage program using natural language processing (NLP) to analyze patient narratives, referrals, and questionnaires (International Patent System (IPS); PCT/US22/43514). The system recommends care levels and disorder-specific digital psychotherapy, enabling data-driven prioritization.^25^ A transformer-based classifier trained on 5,000+ public forum narratives (e.g., Reddit) achieved 90% accuracy in identifying symptomatic phrases compared to experts.^26,27^ The algorithms have also analyzed data from 15+ clinical trials (∼2,000 patients) to predict outcomes based on textual data, care feedback, symptom scores, and engagement.^24,25,28–41^ Key variables (e.g., text composition, severity) allow the system to predict dropout with 70% accuracy four weeks ahead and adjust treatment recommendations.^26^ A decision-making algorithm, using PHQ-9 scores and completion probability, supports personalized care,^42^ suggesting low-intensity care for PHQ-9 < 19 and high completion likelihood, and high-intensity care for severe symptoms and/or low engagement.^26^

This study evaluated an AI-assisted triage system and its impact on reducing wait times in ambulatory psychiatric care.^43–46^ While system validity is important, the primary goal was to assess whether integration improves access to care. By examining wait times and triage accuracy, this study offers a real-world look at streamlining psychiatric referrals. AI-assisted triage may also support specialized treatment pathways and enhance care delivery.

## Materials and Methods

### Participants and Recruitment

Participants were outpatients on the Mental Health and Addiction Care General Stream waitlist at KHSC (HDH site), referred between January 1 and September 30, 2023. This stream serves adults (18+) in Kingston, Frontenac, Lennox & Addington (KFL&A) with mild to severe mental health concerns not requiring specialized, multidisciplinary care (e.g., early psychosis, neuropsychiatry, eating disorders, geriatric). The program ran from October 2023 to March 2024 under a six-month funding period. This timeline avoided disrupting regular triage and focused on recent referrals for accurate data.

### Inclusion and Exclusion Criteria

Inclusion criteria included adult patients on the outpatient waitlist for KHSC's Mental Health and Addiction Care, General Stream (January 1 to September 30, 2023), residing in Ontario, proficient in English, and with reliable internet access to complete the AI-assisted triage and digital psychotherapy programs (accessible, electronic-reader-compatible platform). Participants were informed that this was not a crisis resource, and therapists were not immediately available. Those in a mental health emergency were not enrolled but directed to emergency resources (e.g., ED access, crisis lines), with incidents reported to the principal investigator (PI), a psychiatrist. Participants’ health and comfort were prioritized, and they could withdraw anytime while remaining on the waitlist. If a crisis arose during the program, they would be promptly seen by a psychiatrist.

### Ethics Approval

This project received approval from the Queen's University Health Sciences and Affiliated Teaching Hospitals Research Ethics Board (File: 6041568).

### Procedure

**Figure 1.**
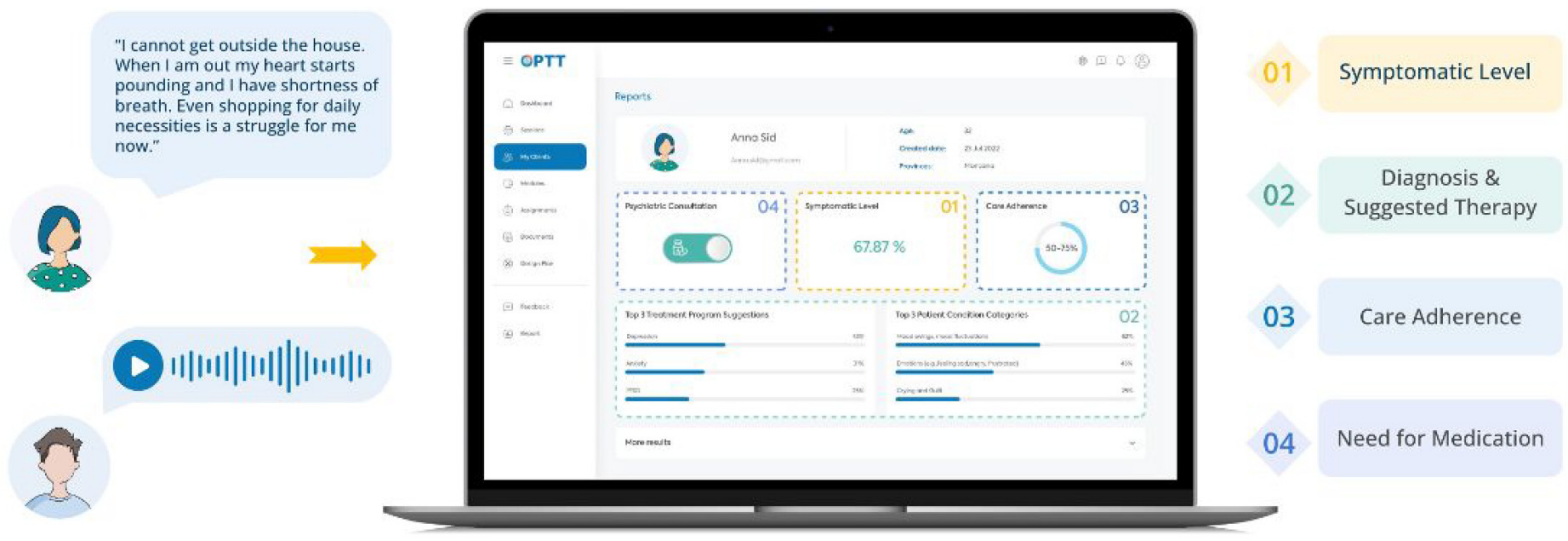
In the triage module, the patient is prompted to explain their mental health challenges using text or voice. Based on their answers, OPTT's analytic algorithm assesses the most sensitive and symptom-related content, facilitating the review process for clinicians. Based on the nature of the problems explained in the patient's narrative, the algorithm provides (1) a report about how symptomatic the patient is, (2) suggests an appropriate disorder-specific digital psychotherapy program, (3) provides a prediction on the patient's adherence to care (i.e., care completion probability), and (4) the suggested care intensity level (4).

Participants were phoned by a research coordinator to assess their interest. If interested, verbal consent was obtained, and participants were sent a link to create an account on the secure cloud-based digital mental health platform, Online Psychotherapy Tool (OPTT).^47,48^ Clients then completed the AI-assisted triage module on OPTT, which included psychoeducation material explaining the journey of someone with mental health problems and the struggles they might experience. Participants then, through typing or voice input, shared their stories, concerns, and symptoms in detail. The triage module also included questionnaires to assess symptom severity, including the PHQ-9, Generalized Anxiety Disorder-7 Item (GAD-7),^49^ and Ask Suicide Questionnaire (ASQ).^42,49,50^ High-risk participants were identified, and a psychiatrist and nurse were notified and contacted the participant. Additionally, OPTT provided high-risk clients with crisis resources and directed them to the ED. The AI-assisted triage program analyzed the module and produced a report for the clinical team, including the participant's answers, questionnaire scores, and recommended care intensity and psychotherapy module (Figure 1).

**Figure 2.**
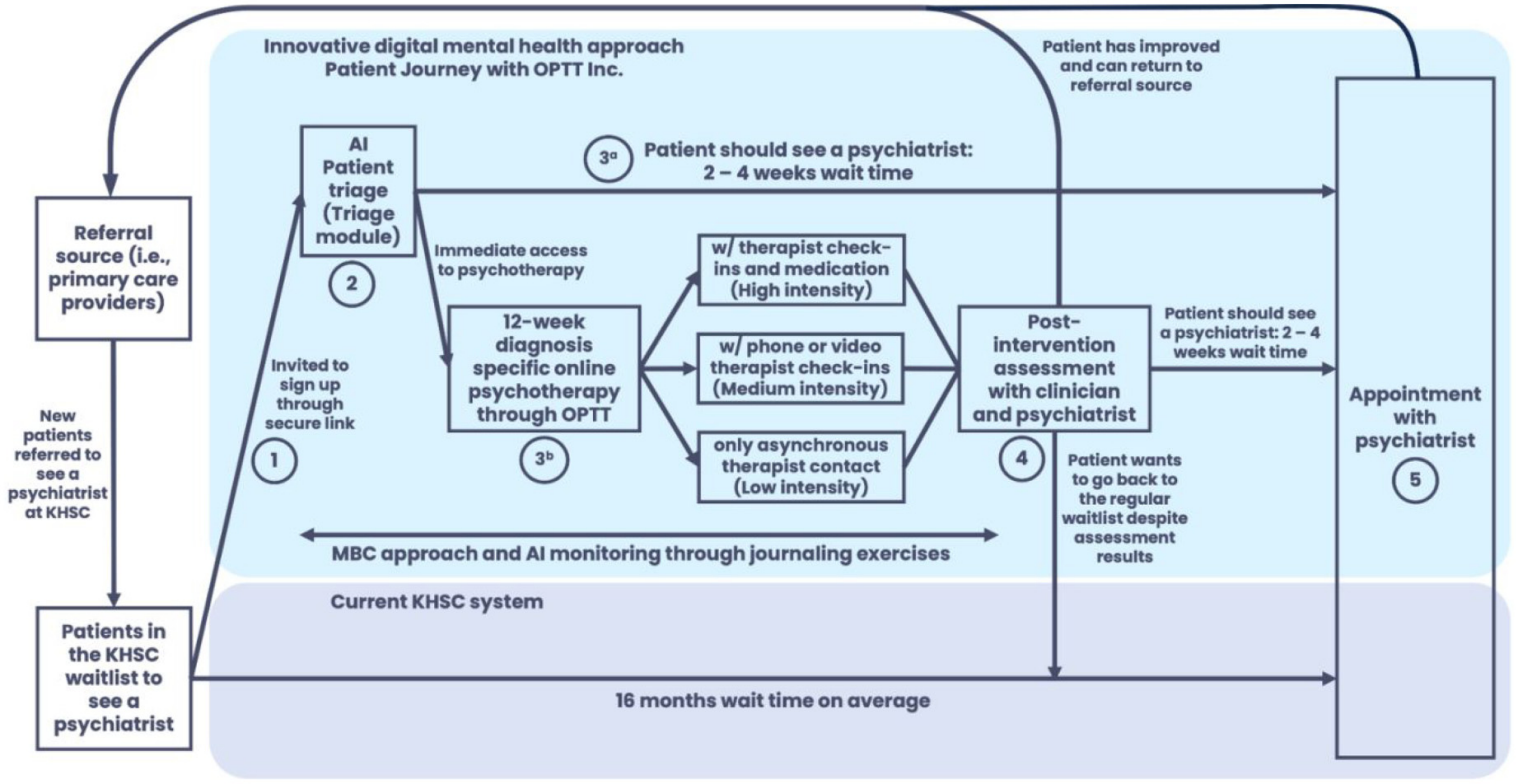
The participant journey from initial referral to psychiatric appointment.

To evaluate the algorithm's decision accuracy, a psychiatrist was used as the human comparator, as there are no established, validated, and evidence-based triage systems to evaluate the decisions. All information from the triage module was reviewed independently by a psychiatrist (before viewing the AI suggestions) to make recommendations for care intensity and psychotherapy programs, based on the Diagnostic and Statistical Manual of Mental Disorders, 5th Edition (DSM-5).^51^ Once the participants’ treatment intensity and psychotherapy modules were confirmed, they were paired with their care team (Figure 2).

### Platform and Technology

OPTT provides a comprehensive range of digital care solutions, including the AI-assisted triage program, which is powered by a proprietary NLP algorithm (IPS; PCT/US22/43514), costing ∼$150/patient/year. Additionally, OPTT also offers online psychotherapy programs for different psychiatric conditions that facilitate data-driven decision-making and streamline care delivery. In this study, the algorithm categorized participants based on symptom severity into four groups:

1. *Mild (Level 1):* Scheduled to see a psychiatrist within six months.
2. *Moderate (Level 2)*: Scheduled to see a psychiatrist within three months.
3. *Severe (Level 3)*: Scheduled to see a psychiatrist within one month.
4. *Inconclusive (Level 4)*: The AI-assisted triage program or the psychiatrist could not allocate due to a lack of information from the participant and/or referral notes. Participants were scheduled to see a psychiatrist within one month and then allocated.

All participants were offered clinician-supervised, disorder-specific, online psychotherapy programs (∼10–12 weeks) while they awaited their psychiatrist appointments.^24,25,28–41^ This ensured participants received timely care and therapeutic intervention, addressing immediate needs and optimizing care delivery. The type of treatment suggested by the AI-assisted triage program was based on symptoms (i.e., generalized anxiety disorder (GAD), major depressive disorder (MDD), dialectical behavioural therapy (DBT), posttraumatic stress disorder (PTSD), insomnia, substance use disorder (SUD), obsessive-compulsive disorder (OCD), depression and anxiety symptoms related to the COVID-19 pandemic). The algorithm suggested multiple modules based on the participant's narrative and reported the probability of their relevance (Figure 1). For this study, the module with the highest probability was used as the AI recommendation.

1. *Mild (Level 1):* Therapist-guided, text-based online psychotherapy.
2. *Moderate and Severe (Levels 2 and 3)*: Therapist-guided, text-based online psychotherapy and a weekly phone/video call.

In some instances, the severity level was categorized as Level 1, but no suitable psychotherapy modality was available to address the participant's specific needs (e.g., attention deficit hyperactivity disorder (ADHD)). Consequently, while their severity level was determined to be Level 1, the psychotherapy module recommendation was inconclusive. These participants were booked for a psychiatric assessment to choose the course of action.

After the program, the therapist and psychiatrist reviewed each participant's progress and questionnaire results. If psychiatric care was still needed, the participant was booked as planned (Figure 2). This decision was based on improvement, program engagement, and whether the referral question was addressed. The therapist communicated the outcome during discharge. A letter outlining progress, compliance, goal achievement, and referral status was sent to the referring clinician. If the clinician had concerns, they could consult the psychiatrist and if an assessment was deemed necessary, the participant was seen to ensure continued access to care.

### Online Psychotherapy

Psychotherapy modules, designed by the PI, an expert in digital psychotherapy,^52^ mirrored their in-person counterparts. The previously validated modules included 20–30 weekly slides of psychotherapeutic concepts and strategies delivered asynchronously.^24,25,28–41^ Participants received one weekly session via OPTT, with instructions to review materials and complete homework exercises to practice techniques and reflect on thoughts and symptoms.^47,48^ Each participant had a designated care provider trained in digital psychotherapy delivery, who reviewed homework and provided personalized text-based feedback. Feedback included validation, progress assessment, strategy suggestions, and key concept summaries. Session-specific feedback templates were used to ensure consistent, efficient feedback while maintaining personalization for each client. Participants and therapists communicated asynchronously via OPTT for non-therapy-related questions, with therapists responding weekly. Technical issues were handled by OPTT's support team.^47,48^ Participants in Levels 2 and 3 had weekly phone/video check-in calls with their therapist. Participants in Level 3 were seen by a psychiatrist who could prescribe medication following standard clinical guidelines.

### Outcomes

The following variables were used to evaluate program efficacy:

1. *Wait times:* Changes in wait times to receive care and therapist time commitment as recorded by the research team.
2. *AI-assisted triage program decision-making accuracy:* Comparing the decision-making of the AI-assisted triage program (treatment intensity and psychotherapy program) to a psychiatrist.
3. *Care sufficiency:* Number of participants in Levels 1 and 2 who no longer required a psychiatric appointment after psychotherapy program completion.

To contextualize the primary outcome of changes in wait time, the system's validity was assessed in parallel by comparing its triage recommendations to those of a psychiatrist. This ensured that observed wait times were interpreted in light of the system's decision-making accuracy.

### Statistical Analysis

All analyses were conducted using IBM SPSS Statistics for Mac, version 25 (IBM Corporation, Armonk, NY, USA), with a two-tailed significance level of *P* < .05. Decision-making accuracy between AI-assisted triage programs and the psychiatrist was evaluated using a contingency table comparing classifications (mild, moderate, severe, inconclusive) and suggested treatments. The proportion of agreement was calculated as the sum of diagonal matches divided by total cases. Chi-square tests assessed whether AI classifications differed from expected distributions under the assumption of no relationship with human classifications. A significant *P*-value indicated a statistically significant association between human and AI classifications. Cramér's *V* measured the strength of these associations.

## Results

### Participants

A total of 294 individuals who had been referred to the KHSC Mental Health Ambulatory Clinic General Stream between January and September 2023 and were on the waitlist were contacted. 83 files were closed due to a lack of response (standard hospital policy: three calls, one email) or no longer residing within Ontario, 13 declined care, and 62 chose not to participate, yielding 136 participants. Of these, five showed interest but did not activate their account, 30 activated their account but did not complete the AI-assisted triage module, and 101 completed the triage (36.05 years [SD = 12.17], 71.74% female). Most were Canadian-born (89.13%), and about half had children (45.65%; number of children = 2.24 (SD = 1.23), age of children = 20.38 years (SD = 9.84)). Those not born in Canada (*n* = 10) immigrated at 16.50 (SD = 15.66) years of age. All participants were fluent in English (Table 1). While 101 participants completed the triage module, 92 completed the demographic information module (Table 1).

**Table 1.** Participant Demographics.

Demographic variable	*n* (%)
Sex (*n* = 92)	
Female	66 (71.74)
Male	26 (28.26)
Gender identity (*n* = 92)	
Female	61 (66.30)
Male	26 (28.26)
Non-binary	3 (3.26)
Other	2 (2.17)
Ethnicity (*n* = 92)	
White	84 (91.30)
Black	2 (2.17)
Indigenous	2 (2.17)
Hispanic	1 (1.09)
Other	3 (3.26)
Marital status (*n* = 92)	
Never married	47 (51.09)
Married	23 (25.00)
Divorced	7 (7.61)
Widowed	3 (3.26)
Other	12 (13.04)
Highest level of education completed (*n* = 90)	
Did not complete secondary school	4 (4.44)
Secondary school diploma	27 (30.00)
College diploma	29 (32.22)
Undergraduate degree	23 (25.56)
Master's degree	5 (5.56)
PhD	2 (2.22)
Employment status (*n* = 92)	
Full-time	36 (39.13)
Unemployed	23 (25.00)
Part-time	6 (6.52)
Student	5 (5.43)
Other	22 (23.91)
Annual income (*n* = 91)	
Under $20,000	32 (35.16)
$20,000–34,999	14 (15.38)
$35,000–49,999	17 (18.68)
$50,000–74,999	12 (13.19)
$75,000–99,999	11 (12.09)
Over $100,000	5 (5.49)

### Wait Time to Receive Care

After completing the triage module, participants were matched with therapists and received their first digital psychotherapy module within 2.64 weeks (SD = 2.72). Level 3 and 4 participants requiring psychiatric assessments were seen within 6.67 weeks (SD = 3.18), while Level 1 and 2 participants needing later psychiatric consultations were seen within 10.90 weeks (SD = 3.18). Before the start of this program, the HDH Ambulatory Mental Health and Addiction Program General Stream had a wait time of ∼seven months, while the overall wait time for all patients referred to the ambulatory clinic, including those directed to other specialized streams, was ∼14 months. By March 2024, the general stream wait time decreased to two months (71.43% improvement), and the overall wait time dropped to 8.48 months (SD = 1.82), a 39.43% improvement.

The AI-assisted triage program optimized patient–clinician interactions. Intake calls averaged 6:23 (SD = 4:56) versus 45 min with a nurse in traditional practice. Digital modules reduced therapist time per patient from one hour in face-to-face sessions to 14:42 (SD = 4:09) for Level 1 participants, with additional weekly check-ins averaging 12:12 (SD = 5:13) for Levels 2 and 3.

### AI-Assisted Triage Program Decision-Making Accuracy

**Figure 3.**
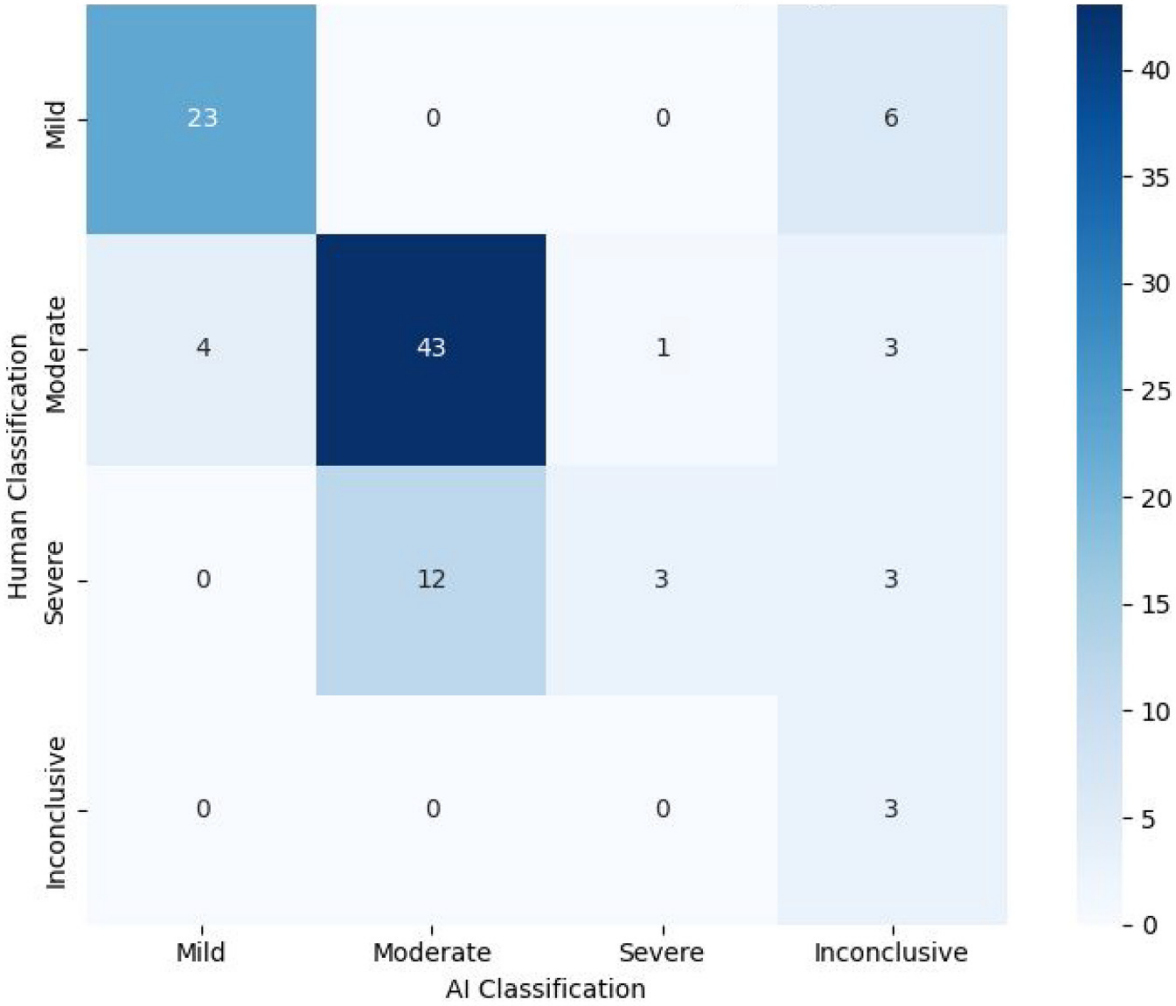
Heat map/crosstab displaying the treatment intensity decision-making of the AI-assisted triage program versus the psychiatrist.

The AI-assisted triage program categorized participants as mild (27/101; 26.73%), moderate (55/101; 54.45%), severe (4/101; 3.96%), and inconclusive (15/101; 14.85%) (Figure 3). It matched psychiatrist classifications for 72/101 (71.29%) participants, including mild (23/29), moderate (43/51), severe (3/18), and inconclusive (3/3) cases. A chi-square test showed significant differences in care intensity classifications (*X*²(9) = 95.9, *P* < .0001), with Cramér's *V* (*V* = 0.56) indicating a moderate-to-strong association. However, 12 cases classified as severe by psychiatrists were rated as moderate by the program, suggesting conservative severity assessments.

**Figure 4.**
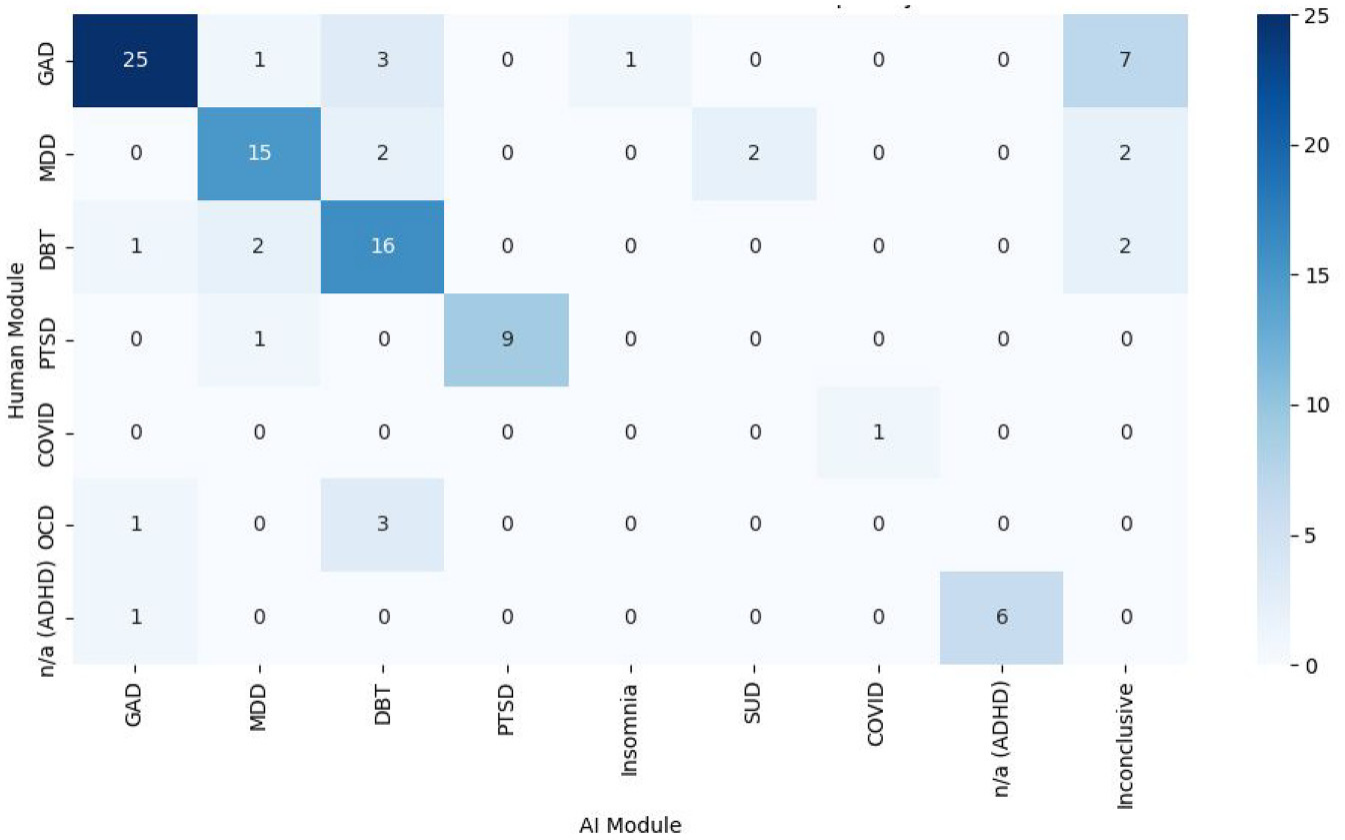
Heat map/crosstab displaying the disorder-specific digital psychotherapy module decision-making of the AI-assisted triage program versus the psychiatrist.

Psychotherapy module recommendations also aligned for 72/101 (71.29%) participants, with a strong agreement for GAD, MDD, DBT, PTSD, COVID-19, and ADHD modules (Figure 4). A chi-square test showed significant classification differences (*X*²(48) = 389, *P* < .0001), with Cramér's *V* (*V* = 0.8) indicating a moderate-to-strong association. Notably, 7/37 (18.92%) GAD cases were labelled inconclusive by the program, and 0/4 OCD cases were classified correctly, with 3/4 (75.00%) misclassified as DBT. These results suggest challenges in distinguishing GAD and OCD features.

### Psychiatric Consultations

24.14% (7/29) and 40.00% (22/50) from Levels 1 and 2 required psychiatric consultation later, resulting in 63.29% of Level 1 and 2 patients not needing to see a psychiatrist. Additionally, all patients flagged for high risk (*n* = 3) by the clinical team were also flagged by the AI-assisted triage program.

## Discussion

The AI-assisted triage program reduced wait times by streamlining decisions, increasing system capacity with digital psychotherapy, and optimizing resources. It enabled efficient, coordinated mental healthcare through scalable digital solutions, instantly assessing multiple patients simultaneously. The program prioritized patients requiring psychiatrist access while directing others to online psychotherapy. Its online format further improved accessibility, scalability, and convenience.

The AI-assisted triage program reduced wait times by +70%, with participants receiving care within 2.5 weeks of completing the triage module. Severe and inconclusive cases saw a psychiatrist within an average of 6.5 weeks, longer than the 4-week target but still a substantial improvement. Mild and moderate cases needing psychiatric care were seen within 11 weeks, meeting the 3–6-month target. Notably, +60% of mild and moderate cases did not require psychiatric consultation. Psychiatrist appointments were reserved for critical cases, while others accessed immediate online psychotherapy, often sufficient for their needs.

The AI-assisted triage program showed strong agreement with the psychiatrist (>70%), demonstrating its reliability in recommending care intensity and disorder-specific psychotherapy. These findings suggest that AI-assisted triage systems can enhance mental healthcare by improving accessibility and optimizing resources. Additionally, when trained on diverse and inclusive data sets, AI-assisted triage has the potential to support equitable care delivery.^53^ While using a psychiatrist as a comparator provided a robust benchmark, it is important to note that triage decisions are inherently subjective. Variability among clinicians would likely occur if multiple experts were involved, highlighting the value of a standardized, evidence-based system.^54^ Prior research showed a model distinguishing symptomatic from asymptomatic sentences achieved 74% accuracy, comparable to a 76% inter-rater overlap between two human experts.^26^ This highlights the potential of AI-assisted triage to reduce inconsistencies. Future research plans to include another rater to demonstrate inter-rater reliability.

The AI-assisted triage program exhibited biases in decision-making, underdiagnosing some participants by categorizing those deemed severe by psychiatrists as moderate. These insights will be used to refine the algorithm, underscoring the importance of collaboration between clinicians and AI developers to enhance decision-making tools. While not perfectly accurate, the algorithm ensured patients received some level of care while on the waitlist, preferable to no care at all. Additionally, every patient was assigned a clinician who could reassess and adjust care as needed.

This study did not deeply explore the role of decision confidence in decision-making accuracy. Preliminary analysis indicated that when the top suggestion had a probability >40%, the algorithm's accuracy exceeded 90%, occurring in 65% of cases. This suggests that decision-making accuracy could improve if the algorithm were used as a supportive tool. Clinicians could rely more on high-confidence outcomes while directing low-confidence cases for further evaluation.

The real-world clinical setting where this project was carried out enhances the generalizability of the findings and demonstrates the feasibility of such systems. However, several limitations should be noted. The lack of long-term follow-up data limits the ability to assess the program's sustainability and lasting effects on patient outcomes. Furthermore, the findings do not have the strength of those that would have been obtained by a randomized design. Selection bias may have impacted the results, as voluntary participation could have led to a sample not fully representative of the broader population. As a novel care delivery model, participants may have faced technical challenges or trust issues with an AI-enabled solution. To address this, a qualitative investigation is underway to explore the challenges, barriers, and perceptions of participants regarding AI in mental healthcare.^55^

Despite these limitations, this study provides a foundation for future investigations into AI-assisted triage in mental healthcare. Several areas warrant further exploration. First, assessing the scalability requires evaluating its interoperability with existing electronic health records, cost-effectiveness, and regulatory compliance. Additionally, understanding the perspectives of patients and providers is crucial, as their trust, acceptance, and engagement with AI will impact its adoption. Moreover, long-term outcomes, including patient quality of life, functional outcomes, well-being, changes in ED utilization, length of hospital stay, rate of readmission, and costs of managing chronic conditions, are crucial to understanding. Finally, replicating this implementation in diverse settings and populations is necessary to examine the generalizability.

A key distinction of this study is its focus on real-world implementation of AI-assisted triage, rather than standalone validation. While validity remains important, the primary goal was to assess whether the system improved timely access to psychiatric care. Analyzing triage accuracy alongside wait times ensured that observed improvements weren’t confounded by inaccurate recommendations.

Future research should also include a comprehensive financial analysis to guide healthcare system decisions. The cost of the proprietary algorithm and access to the OPTT platform is ∼$150/patient/year. Untreated mental health issues impose substantial costs, with healthcare utilization costs being 3.5 times higher for patients with depression, for example, versus those without ($10,064 vs. $2,832) and social service costs being triple ($1,522 vs. $510).^56,57^ Overall, mental health problems can add an average annual cost of $8,244 per person (SD = 40,542).^56,57^ Additionally, unemployment represents the highest per capita cost, with an annual employment loss valued at $32,750 per person.^56,57^ Through this project, wait times were shortened by five months, saving $17,080/per person ($3,435 in healthcare, $13,645 in productivity). In total, this project (*n* = 101) potentially saved the Canadian economy $1,725,080.

As AI advances, ethical concerns like privacy, data security, and bias must be prioritized to build trust and integrate AI solutions into mental healthcare. Future research must continue to ensure that these algorithms are trained on diverse, representative datasets. Moreover, research should explore the benefits and limitations of AI-assisted triage, supporting broader implementation and improved patient care.

## Conclusion

This initiative evaluated the effectiveness of an AI-assisted triage program in reducing wait times and delivering personalized, patient-centred psychiatric care. Online psychotherapy programs were offered as accessible, scalable, cost-effective, and validated treatments, enhancing patients’ quality of life. AI-assisted triage enabled standardized, high-quality care while maximizing system capacity. At a system level, it demonstrated the potential to reduce wait times and improve equitable, affordable care access. By enhancing capacity, streamlining workflows, and supporting clinical decision-making, this model addresses key challenges in care delivery. Additionally, it may reduce ED strain, improve chronic disease management by addressing comorbidities, and establish a foundation for measurement-based care by integrating evidence-based metrics to optimize outcomes.
